# Supplementary material for: PDK1 Inhibitor BX795 Improves Cisplatin and Radio-Efficacy in Oral Squamous Cell Carcinoma by Downregulating the PDK1/CD47/Akt-Mediated Glycolysis Signaling Pathway
Source: Int J Mol Sci. 2021 Oct 25;22(21):11492. doi: 10.3390/ijms222111492 (PMC8584253; doi:10.3390/ijms222111492)
Supplement: Supplementary file 1 [file ijms-22-11492-s001.zip › ijms-1411830-supplementary.pdf]

SUPPLEMENTARY INFORMATION

PDK1 inhibitor BX795 improves cisplatin and radio-efficacy in oral squamous cell carcinoma by downregulating the PDK1/CD47/Akt-mediated glycolysis signalling pathway

Shin Pai, MD[1,2], Vijesh Kumar Yadav, PhD[3,4], Kuang-Tai Kuo, MD., PhD [5,6], Narpati Wesa Pikatan, PhD[3,4,] Chun-Shu Lin, MD., PhD [7], Ming-Hsien Chien, PhD[1], Wei-Hwa Lee, MD., PhD[3,8], Michael Hsiao, PhD[9], Shao-Chih Chiu[10,11,12], Chi-Tai Yeh, PhD[1,3,4*] and Jo-Ting Tsai, MD., PhD[1,13,14*]

Authors' affiliation:

1 Graduate Institute of Clinical Medicine, College of Medicine, Taipei Medical University, Taipei 110, Taiwan.

2 Department of Oral & Maxillofacial Surgery, Saint Martin de Parres Hospital, Chiayi City 60069, Taiwan;

3 Department of Medical Research and Education, Taipei Medical University - Shuang Ho Hospital, New Taipei City 23561, Taiwan.

4 Department of Medical Laboratory Science and Biotechnology, Yuanpei University of Medical Technolo-gy, Hsinchu City 30015, Taiwan.

5 Division of Thoracic Surgery, Department of Surgery, School of Medicine, College of Medicine, Taipei Medical University, Taipei 110, Taiwan

6 Division of Thoracic Surgery, Department of Surgery, Shuang Ho Hospital, Taipei Medical University, New Taipei City 23561, Taiwan

7 Department of Radiation Oncology, Tri-Service General Hospital, National Defense Medical Center, Tai-pei, Taiwan;

8 Department of Pathology, Taipei Medical University - Shuang Ho Hospital, New Taipei City 23561, Taiwan.

9 Genomics Research Center, Academia Sinica, Taipei, Taiwan;

10 Graduate Institute of Biomedical Sciences, China Medical University, Taichung 40447, Taiwan.

11 Translational Cell Therapy Center, Department of Medical Research, China Medical University Hospital, Taichung 404, Taiwan

12 Drug Development Center, China Medical University, Taichung 40447, Taiwan

13 Department of Radiology, School of Medicine, College of Medicine, Taipei Medical University, Taipei, Taiwan;

14 Department of Radiology, Taipei Medical University - Shuang Ho Hospital, New Taipei City, Taiwan.

* Correspondence: author(s):

* Correspondence: Prof. Chi-Tai Yeh, PhD., Department of Medical Research and Education, Taipei Med-ical University - Shuang Ho Hospital, New Taipei City 23561, Taiwan; Tel: +886-2-2490088 ext. 8881, Fax: +886-2-2248-0900, E-mail: ctyeh@s.tmu.edu.tw

* Correspondence: Prof. Jo-Ting Tsai, MD., PhD., Department of Radiation Oncology, Cancer Center, Taipei Medical University - Shuang Ho Hospital, New Taipei City 23561, Taiwan; Tel: +886-2-2490088 ext. 8885, Fax: +886-2-2248-0900, E-mail: 10576@s.tmu.edu.tw

Working title: PDK1 /CD47/Akt-mediated glycolysis signalling in OSCC

Supplementary Table S1. Western blot antibodies sheet.

No.	Target	Dilution	Catalog	kDa
1	GAPDH	1:1000	GAPDH (0411K) Mouse mAb SC-27724	37
2	CD47	1:500	CD47 (B6H12) Mouse mAb SC-12730	47
3	p-PI3K	1:1000	Phospho-PI3 Kinase p85 (Tyr458)/p55 (Tyr199) Antibody #4228	60 and 85
4	PI3K	1:1000	PI3 Kinase p110α (C73F8) Rabbit mAb #4249	110
5	p-PDK1	1:1000	Phospho-PDK1 (Ser241) Antibody #3061	58 - 68
6	PDK1	1:1000	PDK1 (D37A7) Rabbit mAb #5662	58 - 68
7	p-AKT	1:1000	Phospho-Akt (Ser473) (D9E) XP® Rabbit mAb #4060	60
8	AKT	1:1000	Akt Rabbit Polyclonal antibody #9272	60
9	p-mTOR	1:1000	Phospho-mTOR (Ser2481) Antibody #2974	289
10	mTOR	1:1000	mTOR (7C10) Rabbit mAb #2983	289
11	Sox2	1:1000	Sox2 (L1D6A2) Mouse mAb #4900	35
12	Oct-4	1:1000	Oct-4 (C30A3) Rabbit mAb #2840	45
13	CD133	1:1000	CD133 Rabbit mAb 18470-1-AP	120
14	LDHA	1:1000	LDHA (C4B5) Rabbit mAb #3582	37
15	Bak	1:1000	Bak (D2D3) Rabbit mAb #6947	25
16	Bax	1:1000	Bax (D2E11) Rabbit mAb #5023	20
17	Bcl-2	1:1000	Bcl-2 (50E3) Rabbit mAb #2870	26
18	E-Cadherin	1:1000	E-Cadherin (24E10) Rabbit mAb #3195	125-135
19	N-cadherin	1:1000	N-Cadherin Rabbit polyclonal Antibody #4061	140
20	Vimentin	1:1000	Anti-Vimentin antibody (ab137321)	57
21	Slug	1:1000	Slug (C19G7) Rabbit mAb #9585	30
22	Snail	1:1000	Snail Rabbit mAb #3879P	29
23	γ-H2AX	1:1000	Gamma-H2AX Recombinant Rabbit Monoclonal Antibody (BLR053F)	15
24	H2AX	1:1000	Histone H2A.X (D17A3) XP® Rabbit mAb #7631	15
25	Cleaved Caspase-3	1:1000	Cleaved Caspase-3 (Asp175) Antibody #9661	17.19
26	Cleaved Caspase-9	1:1000	Cleaved Caspase-9 (Asp315) Antibody (Human Specific) #9505	35
27	β-Catenin	1:1000	β-Catenin (6B3) Rabbit mAb #9582	92
28	PFKP	1:1000	PFKP (D2E5) Rabbit mAb #12746	80
29	PDK3	1:1000	PDK3 Polyclonal antibody (12215-1-AP)	47

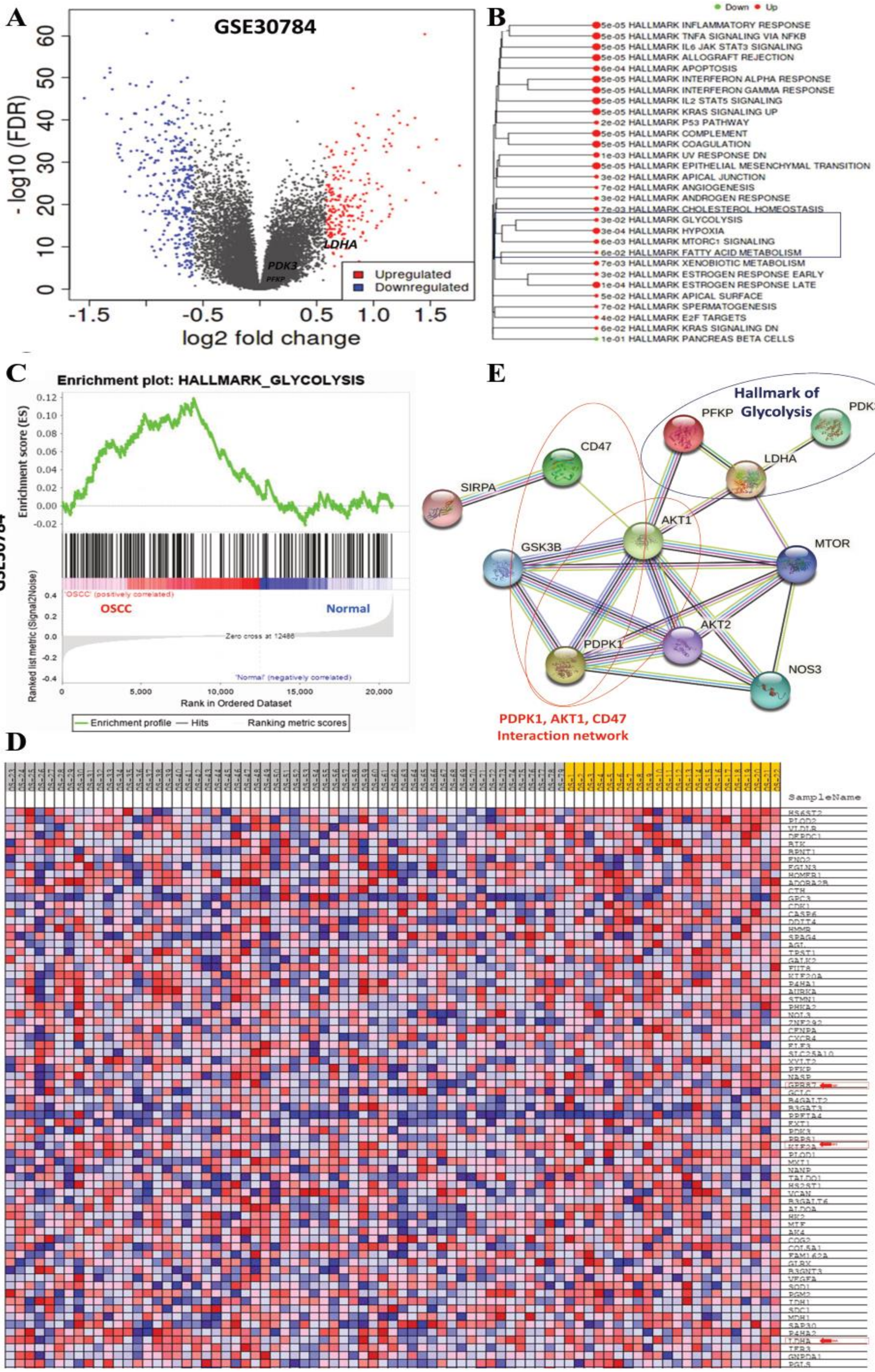
A
GSE30784
-log10 (FDR)
log2 fold change
LDHA
PDK3
PFKP
Upregulated
Downregulated
B
Down
Up
5e-05 HALLMARK INFLAMMATORY RESPONSE
5e-05 HALLMARK TNFA SIGNALING VIA NFKB
5e-05 HALLMARK IL6 JAK STAT3 SIGNALING
5e-05 HALLMARK ALLOGRAFT REJECTION
6e-04 HALLMARK APOPTOSIS
5e-05 HALLMARK INTERFERON ALPHA RESPONSE
5e-05 HALLMARK INTERFERON GAMMA RESPONSE
5e-05 HALLMARK IL2 STAT5 SIGNALING
5e-05 HALLMARK KRAS SIGNALING UP
2e-02 HALLMARK P53 PATHWAY
5e-05 HALLMARK COMPLEMENT
5e-05 HALLMARK COAGULATION
1e-03 HALLMARK UV RESPONSE DN
5e-05 HALLMARK EPITHELIAL MESENCHYMAL TRANSITION
3e-02 HALLMARK APICAL JUNCTION
7e-02 HALLMARK ANGIOGENESIS
3e-02 HALLMARK ANDROGEN RESPONSE
7e-03 HALLMARK CHOLESTEROL HOMEOSTASIS
3e-02 HALLMARK GLYCOLYSIS
3e-04 HALLMARK HYPOXIA
6e-03 HALLMARK MTORC1 SIGNALING
6e-02 HALLMARK FATTY ACID METABOLISM
7e-03 HALLMARK XENOBIOTIC METABOLISM
3e-02 HALLMARK ESTROGEN RESPONSE EARLY
1e-04 HALLMARK ESTROGEN RESPONSE LATE
5e-02 HALLMARK APICAL SURFACE
7e-02 HALLMARK SPERMATOGENESIS
4e-02 HALLMARK E2F TARGETS
6e-02 HALLMARK KRAS SIGNALING DN
1e-01 HALLMARK PANCREAS BETA CELLS
C
Enrichment plot: HALLMARK_GLYCOLYSIS
GSE30784
Enrichment score (ES)
Ranked list metric (Signal2Noise)
'OSCC' (positively correlated)
OSCC
Normal
Zero cross at 12486
'Normal' (negatively correlated)
Rank in Ordered Dataset
Enrichment profile
Hits
Ranking metric scores
E
Hallmark of Glycolysis
PFKP
PDK3
LDHA
CD47
SIRPA
AKT1
GSK3B
MTOR
AKT2
PDPK1
NOS3
PDPK1, AKT1, CD47 Interaction network
D
SampleName

Supplementary Figure S1. Identification of differentially expressed genes (DEGs) in gene expression omnibus (GEO) datasets. (A) Volcano plot of DEGs in GSE30784., Red, blue and gray color represent the relatively high, low and equal expression of genes in the corresponding group, respectively. $p < .05$ and $|log(FC)| >1$ were set as the threshold. (B-D) Gene Ontology (GO) and Gene Set Enrichment Analysis (GSEA) of OSCC tumor samples with the normal sample. (E) STRING database analysis of the relationship between PDK1, Akt, CD47, LDHA genes. Network nodes represent proteins encoded by genes. * $p < 0.05$, ** $p < 0.01$, *** $p < 0.001$.

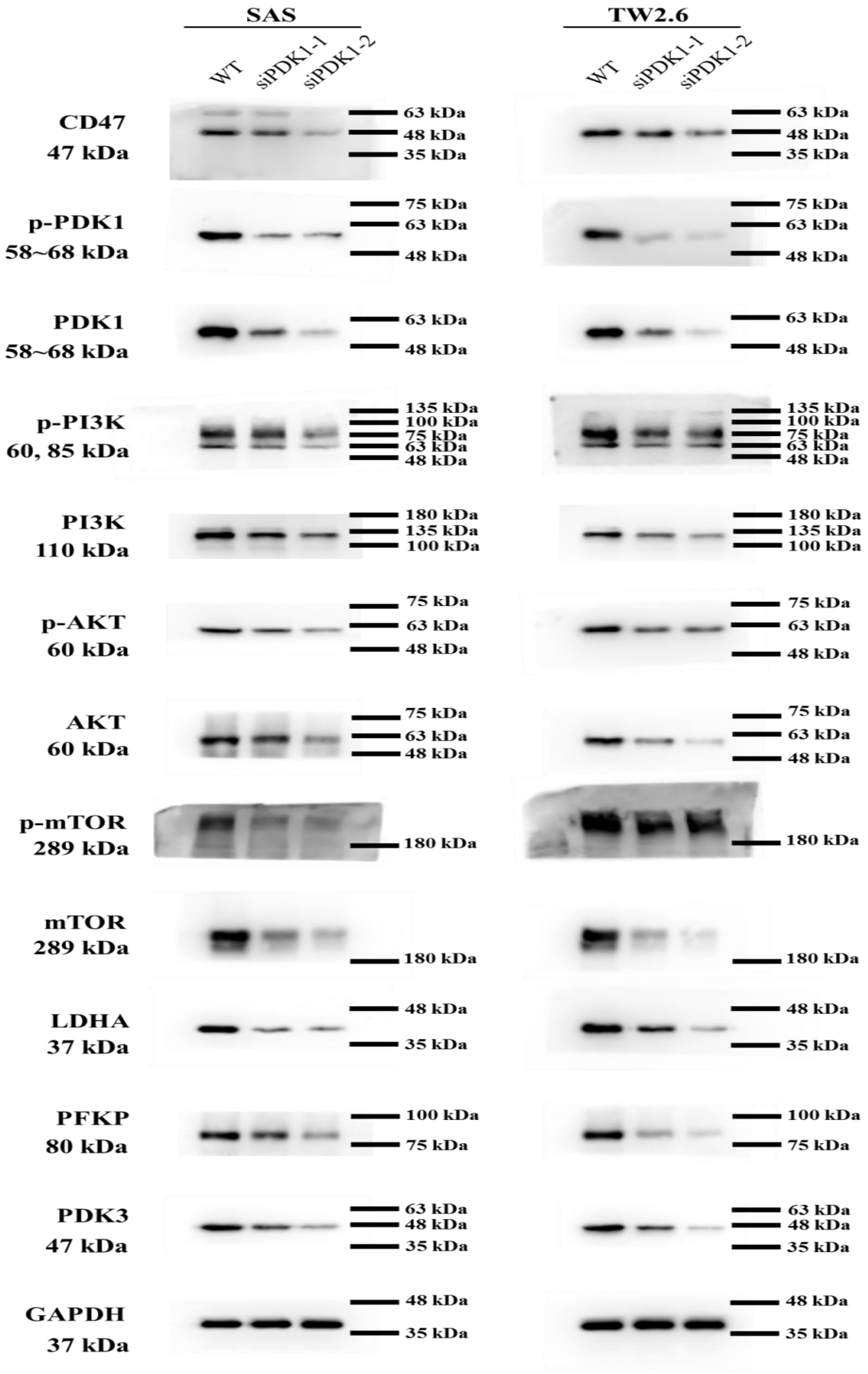

Supplementary Figure S2. Full-size blots of Figure 2D

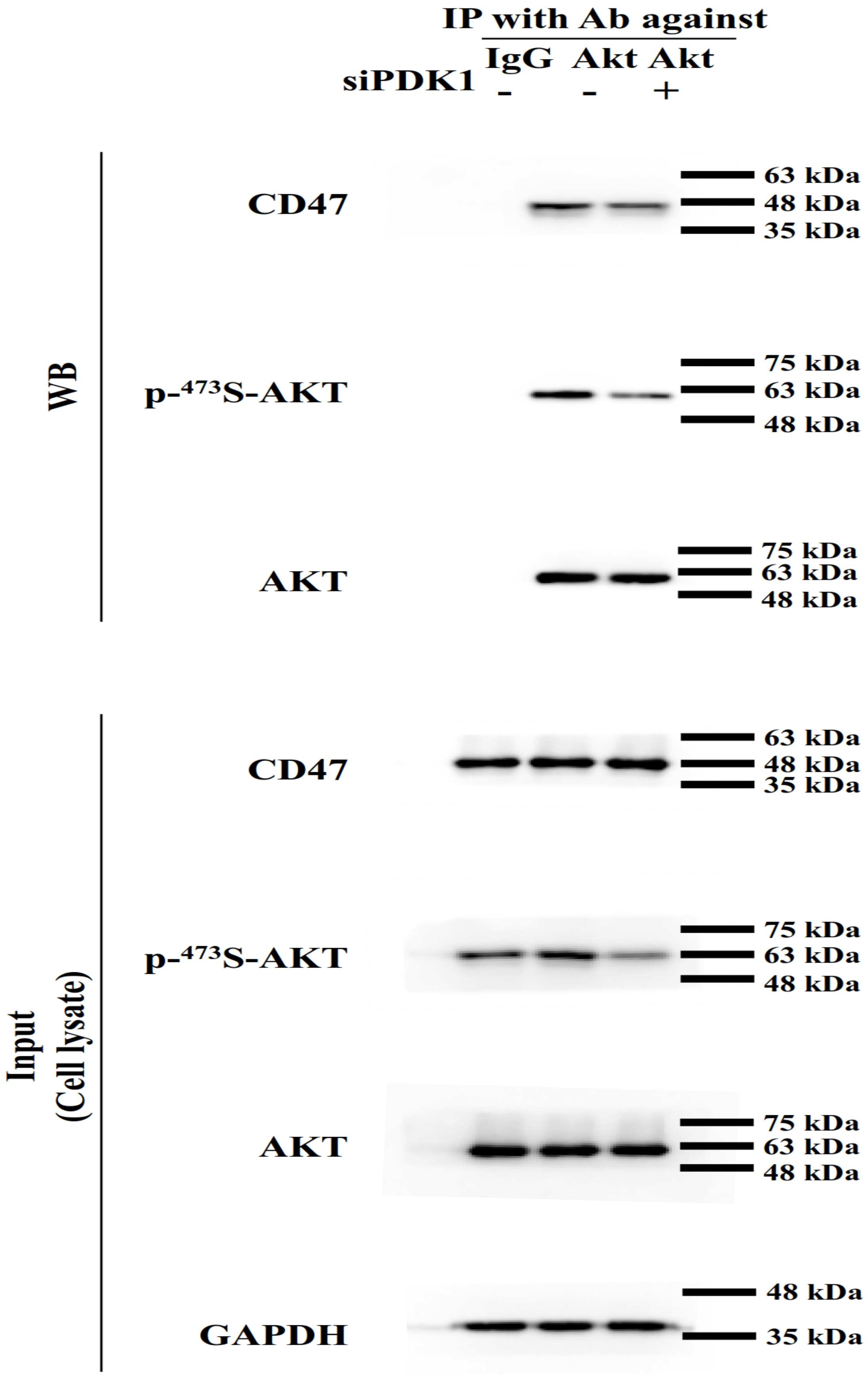

Supplementary Figure S3. Full-size blots of Figure 2E

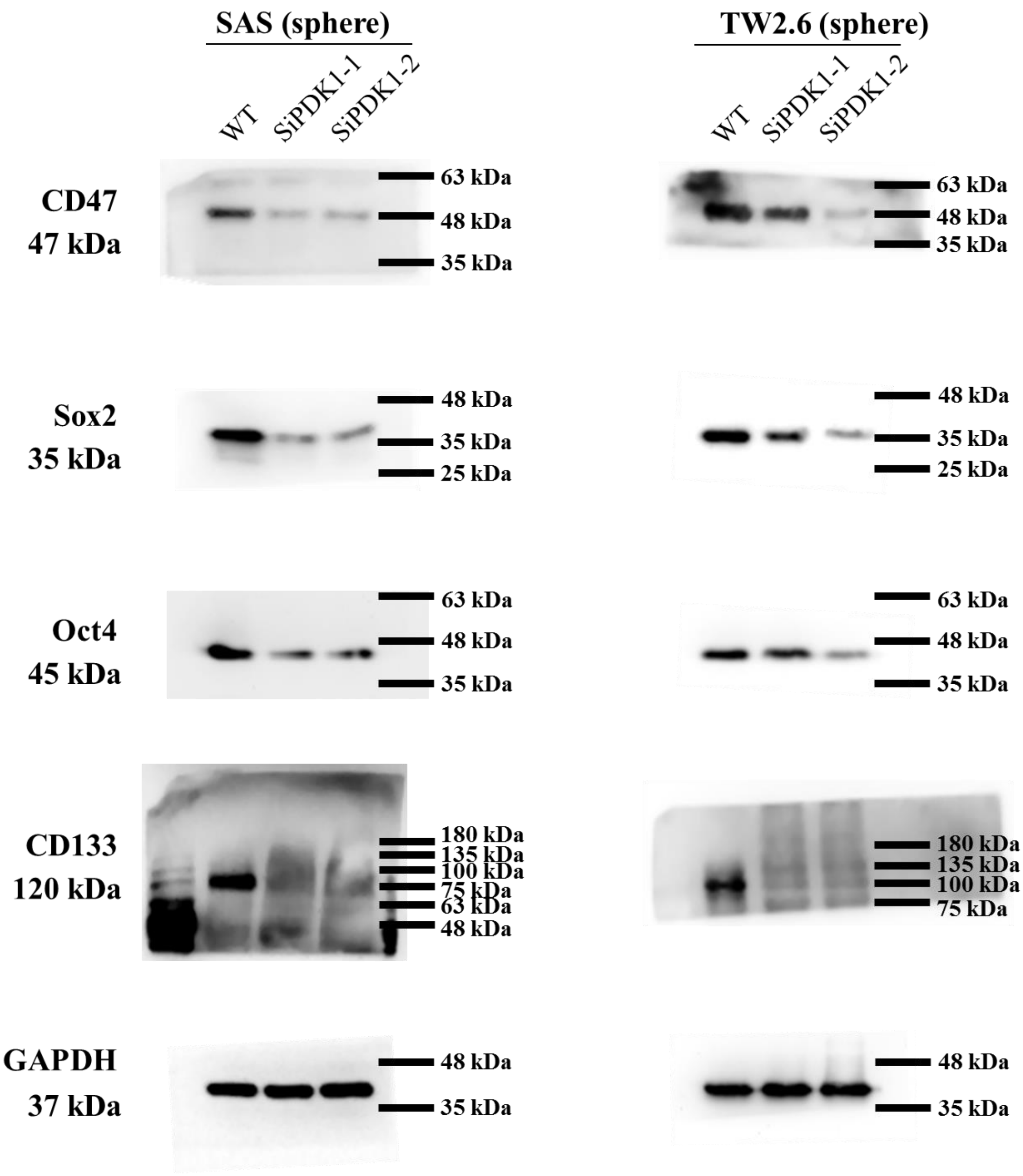

Supplementary Figure S4. Full-size blots of Figure 3D

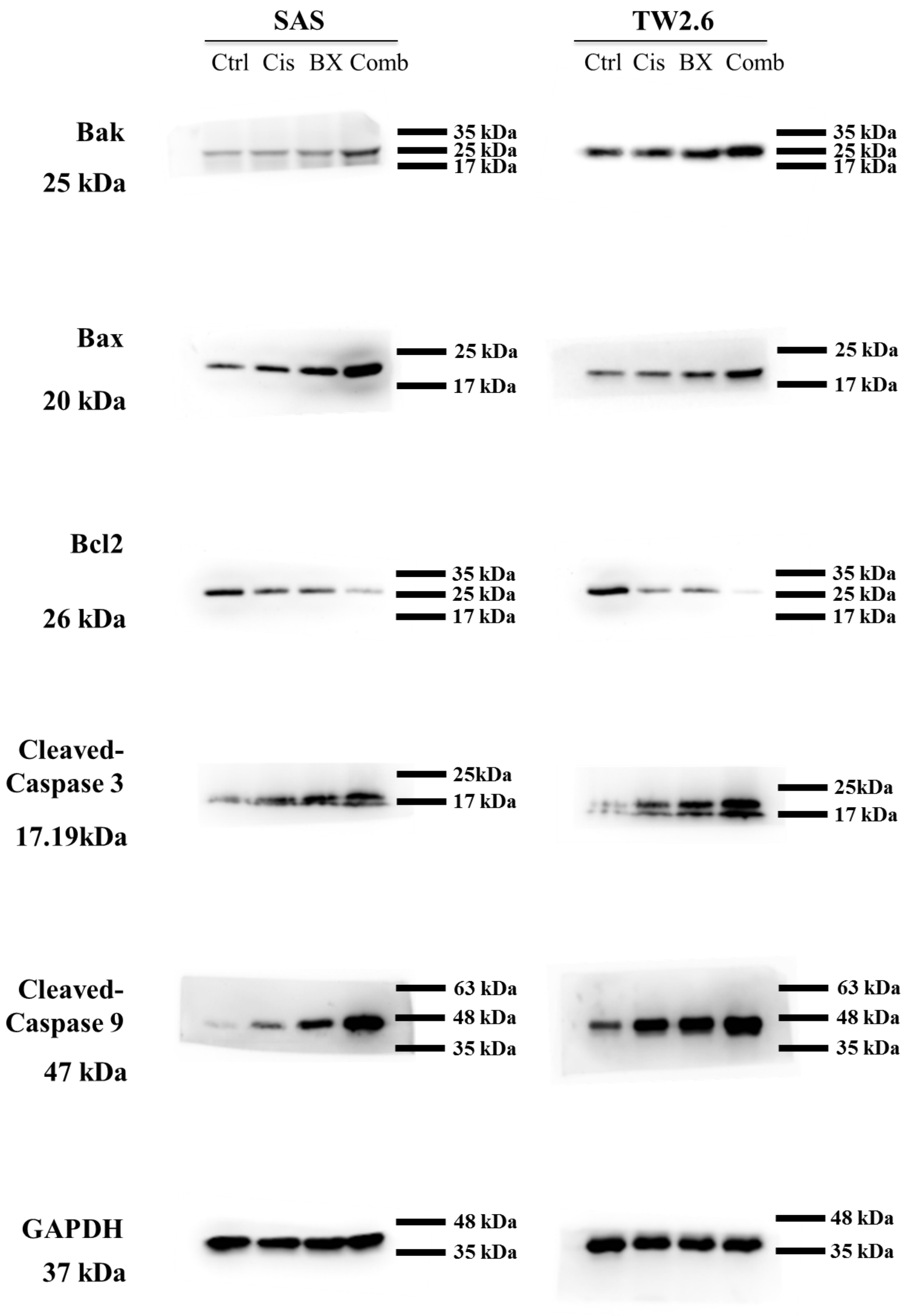

Supplementary Figure S5. Full-size blots of Figure 4D

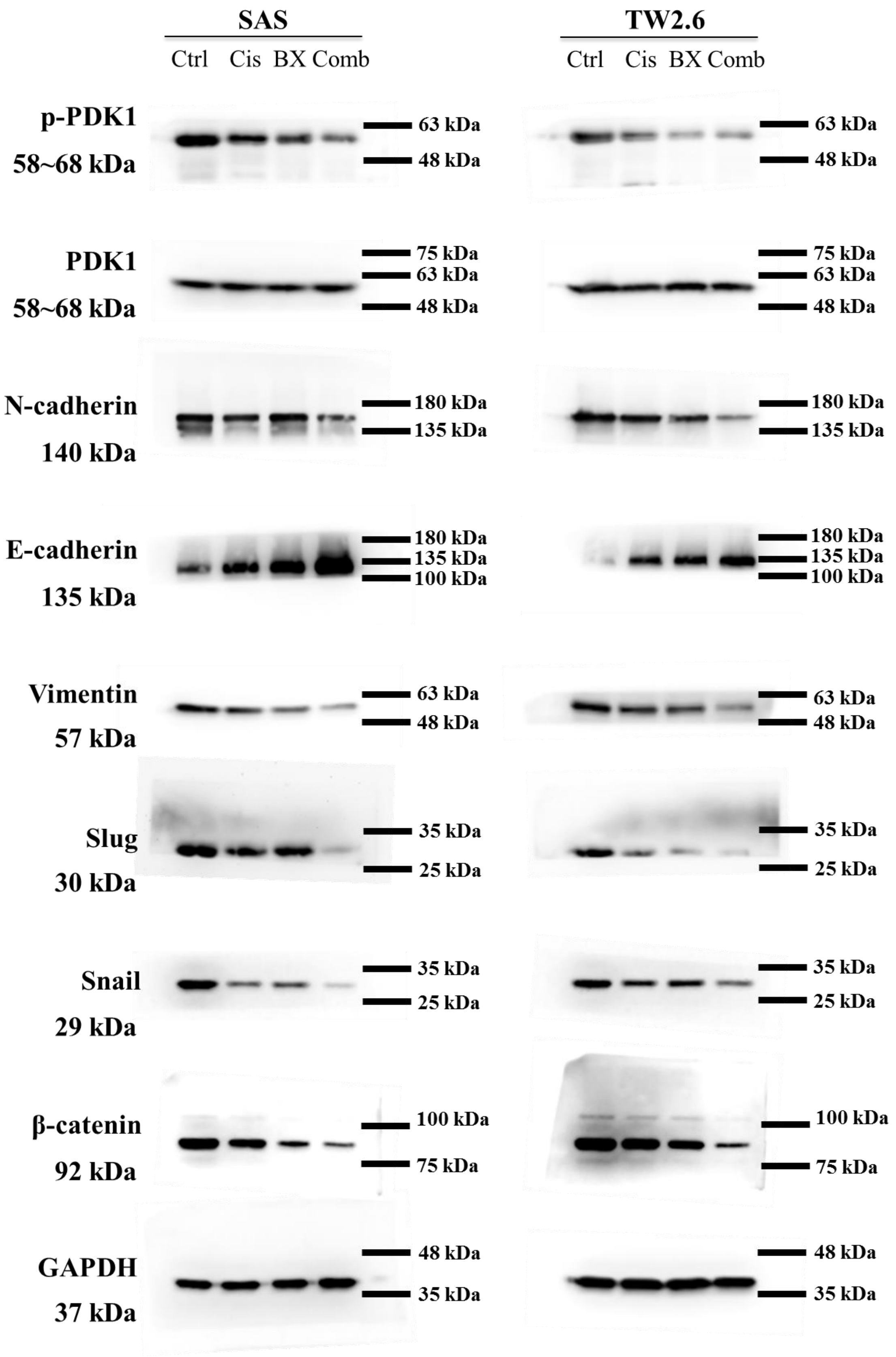

Supplementary Figure S6. Full-size blots of Figure 5D

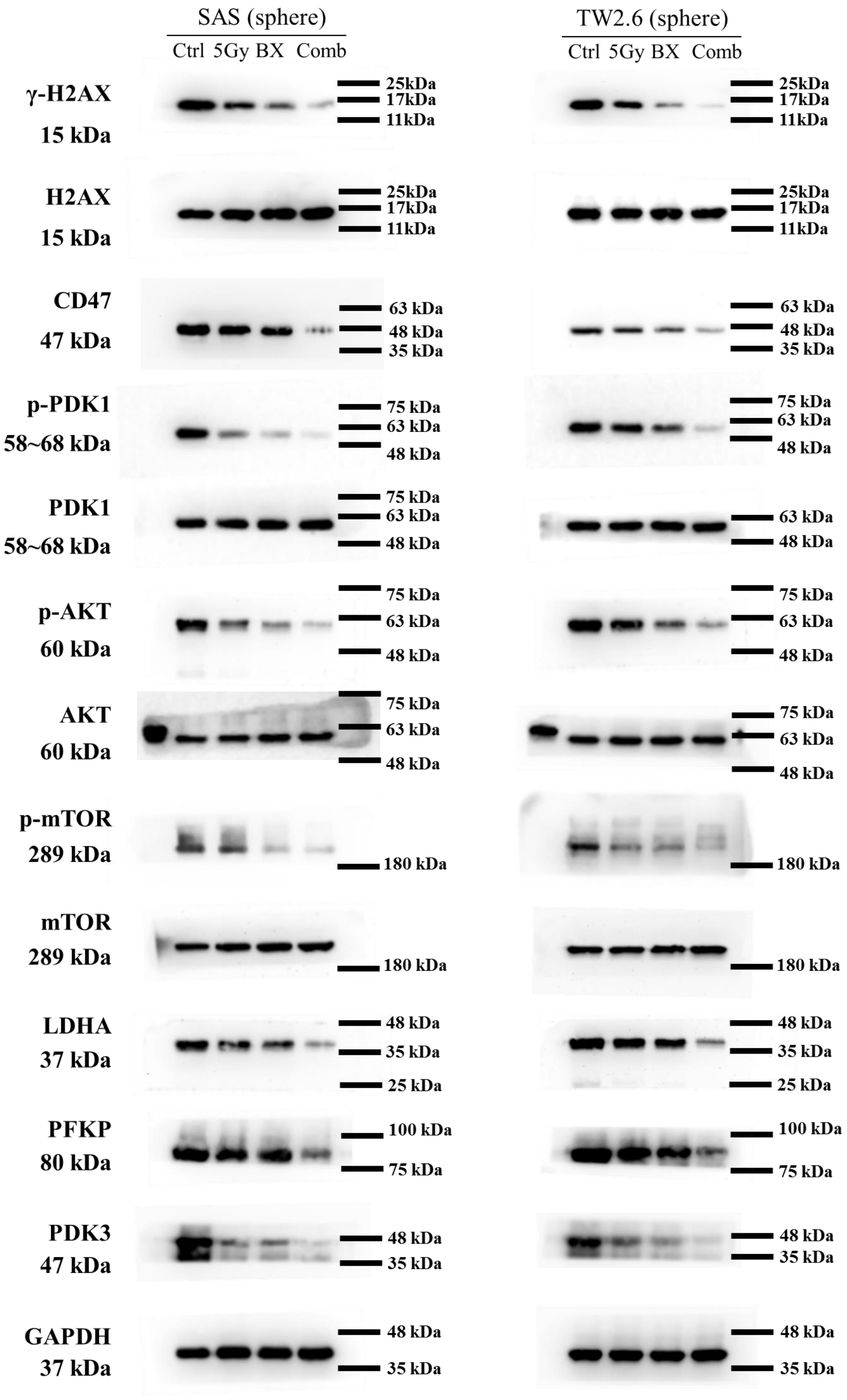

Supplementary Figure S7. Full-size blots of Figure 6F
